# Comparable Autoantibody Serum Levels against Amyloid- and Inflammation-Associated Proteins in Parkinson’s Disease Patients and Controls

**DOI:** 10.1371/journal.pone.0088604

**Published:** 2014-02-21

**Authors:** Walter Maetzler, Anja Apel, Markus Langkamp, Christian Deuschle, Sarah Selina Dilger, Johannes Georg Stirnkorb, Claudia Schulte, Erwin Schleicher, Thomas Gasser, Daniela Berg

**Affiliations:** 1 Center of Neurology, Department of Neurodegeneration and Hertie Institute for Clinical Brain Research, University of Tuebingen, Tuebingen, Germany; 2 DZNE, German Center for Neurodegenerative Diseases, University of Tuebingen, Tuebingen, Germany; 3 Mediagnost, Reutlingen, Germany; 4 Department of Internal Medicine IV, University Hospital Tuebingen, Tuebingen, Germany; UCL Institute of Neurology, United Kingdom

## Abstract

Naturally occurring autoantibodies (NAbs) against a number of potentially disease-associated cellular proteins, including Amyloid-beta_1–42_ (Abeta_1–42_), Alpha-synuclein (Asyn), myelin basic protein (MBP), myelin oligodendrocyte glycoprotein (MOG), and S100 calcium binding protein B (S100B) have been suggested to be associated with neurodegenerative disorders, in particular Alzheimer’s (AD) and Parkinson’s disease (PD). Whereas the (reduced) occurrence of specific NAbs in AD is widely accepted, previous literature examining the relation of these NAb titres between PD patients and controls, as well as comparing these levels with demographic and clinical parameters in PD patients have produced inconsistent findings. We therefore aimed, in a cross-sectional approach, to determine serum titres of the above NAbs in a cohort of 93 PD patients (31 of them demented) and 194 controls. Levels were correlated with demographic and clinical variables, cerebrospinal fluid Abeta_1–42_, total tau and phospho-tau levels, as well as with single nucleotide polymorphisms (SNPs) of genes which either have been reported to influence the immune system, the amyloid cascade or the occurrence of PD (*ApoE, GSK3B, HLA-DRA, HSPA5, SNCA*, and *STK39*). The investigated NAb titres were neither significantly associated with the occurrence of PD, nor with demographic and clinical parameters, neurodegenerative markers or genetic variables. These results argue against a major potential of blood-borne parameters of the adaptive immune system to serve as trait or state markers in PD.

## Introduction

Naturally occurring autoantibodies (NAbs) probably act in eliminating circulating proteins, before they can elicit a damaging response [1], [2]. In the context of neurodegenerative disorders evidence has accumulated that such NAbs also exist which may be involved e.g. in the physiologic clearance of the misfolded proteins [1], [3]. More specifically, evidence accumulates that individuals with increased risk for, and patients with Alzheimer’s disease (AD) have altered levels of NAbs that are directed against Amyloid-beta (Abeta) [4]–[7]. This has recently led to a phase 2 clinical trial with intravenous immunoglobulin in AD patients [8]. The role of NAbs in idiopathic Parkinson’s disease (PD) is less clear. Blood-borne NAbs directed against the putatively most important self-antigen in PD, Alpha-synuclein (Asyn), have been reported to be higher [9], [10], not significantly different [11] and lower [12] compared to respective control cohorts. All the above-mentioned studies used recombinant Asyn as an antigen.

In a previous work, we found higher serum NAb levels against recombinant Abeta_1–42_, Asyn, myelin basic protein (MBP), myelin oligodendrocyte glycoprotein (MOG), and S100 calcium binding protein B (S100B) in 13 demented PD patients as well as in 14 patients with dementia with Lewy bodies (DLB), compared to 33 patients with other dementias and 31 controls [13]. An increased immune response to S100B in PD was also found in another study [14], supporting the view that S100B may be crucially involved in the pathophysiology of PD [15]. Also, higher Asyn NAb levels were very recently found in 19 DLB patients (and in 15 AD patients) compared to 16 control individuals [16]. The role of the above mentioned proteins is well established, but the influence of the corresponding NAbs is not well defined.

As all of the mentioned studies included relatively small cohort sizes, here we tested the utility of serum levels of the above mentioned NAbs as potential markers of presence and severity (including cognitive dysfunction) of PD in a considerably larger cohort of PD patients and controls, using a comprehensive test battery including demographic, clinical and neurodegenerative parameters. Moreover, we evaluated respective NAb titres in association with different single nucleotide polymorphisms (SNPs) of genes which either have been reported to influence the activity of the adaptive immune system, the amyloid cascade or have been associated with the occurrence of PD (*APOE* [17], [18], *GSK3B* [19], [20], *HLA-DRA* [21], [22], *HSPA5* [23], [24], *SNCA* [25]–[27], and *STK39* [28], [29]).

## Materials and Methods

### Ethics Statement

The study was approved by the local ethics committee (Ethics committee of the medical faculty of Eberhard Karls University Tuebingen and of the University Hospital Tuebingen, Tuebingen, Germany), and was performed according to the principles expressed in the Declaration of Helsinki. All participants capable to consent gave their written informed consent. In case of compromised capacity of the participants to consent (MMSE ≤18 points, and/or another person was named to make decisions on behalf of the person), responsible persons gave their written consent on the behalf of the participant.

### Study Participants

Ninety three PD patients were recruited from the ward and the outpatient clinic of the Neurodegenerative Department of the University of Tuebingen. They were diagnosed by specialists in the field of neurodegenerative movement disorders (WM, TG, DB), and fulfilled the UKPDS Brain Bank criteria [30]. Demented PD (PDD) patients also met dementia criteria of the Diagnostic and Statistical Manual of Mental Disorders-IV (DSM-IV). One hundred and ninety-four healthy controls were recruited from the TREND study (www.trend-studie.de) [31], [32]. Any clinical sign for a neurodegenerative disorder in these individuals led to exclusion from the study. Individuals with recent medical history or actual clinical and laboratory signs for inflammation or infection were not included.

Medical history and demographics were obtained from all individuals (sex, age, age at onset parkinsonism and dementia), and all participants underwent clinical testing including a Hoehn & Yahr (H&Y) staging and a Mini-Mental State Examination (MMSE).

**Table 1** and Table 2 provide an overview of demographic, clinical, and biochemical data of the cohorts and subcohorts.

**Table 1:** Demographic, clinical, routine biochemical and genetic data of the cohorts.

	PD	Controls	*p*-values
Individuals (f in %)	93 (38.7)	194 (55.7)	0.007*
Age (years)	70 (44–88)	63 (50–80)	<0.0001*
AAO parkinsonism (years)	62 (39–84)		
Duration parkinsonism (years)	9.5 (1–26)		
H&Y stage (1–5)	2 (1–4)		
BDI (0–63)	9 (1–29)	5.5 (0–40)	0.0007*
MMSE (0–30)	27 (10–30)	29 (27–30)	<0.0001*
UPDRS (0–199)	5 (0–43)	0 (0–7)	<0.0001*
CSF Abeta_1–42_ (pg/ml)	540 (141–1127)		
CSF t-tau (pg/ml)	217 (61–927)		
CSF p-tau (pg/ml)	43 (21–107)		
*ApoE4* (%)	19.35	25.77	0.23
*GSK3B* (A allele of SNPrs6438552)	86.36	82.47	0.65
*HLA-DRA* (A allele of SNPrs3129882)	82.05	73.58	0.14
*HSPA5* (A allele of SNP rs430397)	12.86	15.03	0.66
*SNCA* (G allele of SNP rs356219)	56.41	65.46	0.16
*STK39* (A allele of SNP rs4668049)	31.08	25.4	0.35

Demographic, clinical and biochemical data of patients with Parkinson’s disease (PD) and controls are presented with median (range) or percentage of total. *P*-values were determined using the Wilcoxon rank sum test or the Fisher’s exact test. Aao, age at onset; *ApoE4,* at least one *Apolipoprotein E4* allele; BDI, Beck Depression Inventory; CSF, cerebrospinal fluid; f, female; *GSK3B, Glycogen synthase kinase 3 beta*; *HLA-DRA, Human leucocyte antigen/Major histocompatibility complex, class II, DR alpha chain; HSPA5, Heat shock 70 kDa protein 5*; H&Y, Hoehn & Yahr stage; MMSE, Mini-Mental State Examination; p-tau, phospho-tau; *SNCA, Synuclein alpha; STK39, Serine threonine kinase 39*; t-tau, total tau; UPDRS, Unified Parkinson’s Disease Rating Scale.

**Table 2:** Demographic, clinical, routine biochemical and genetic data of the subcohorts.

	PDND	PDD	*p*-values
Individuals (f in %)	62 (40.3)	31 (35.5)	0.65
Age (years)	68 (44–84)	74 (54–88)	<0.0001*
AAO parkinsonism (years)	60 (39–78)	64 (48–84)	0.008*
Duration parkinsonism (years)	7 (1–23)	11 (1–26)	0.11
Aao dementia (years)		69 (50–80)	
Duration dementia (years)		5 (0–10)	
H&Y stage (1–5)	2 (1–3.5)	2.25 (1–4)	0.24
BDI (0–63)	8.5 (1–21)	9 (2–29)	0.77
MMSE (0–30)	28.5 (19–30)	23 (10–26)	<0.0001*
UPDRS (0–199)	7 (0–37)	3 (0–43)	0.60
CSF Abeta_1–42_ (pg/ml)	750 (269–1127)	419 (141–737)	<0.0001*
CSF t-tau (pg/ml)	195 (109–432)	244 (61–927)	0.18
CSF p-tau (pg/ml)	43 (26–72)	42 (21–107)	0.69
*ApoE4* (%)	22.58	12.9	0.27
*GSK3B* (A allele of SNPrs6438552)	92.86	75.0	0.24
*HLA-DRA* (A allele of SNPrs3129882)	83.33	79.17	0.66
*HSPA5* (A allele of SNP rs430397)	11.11	18.75	0.42
*SNCA* (G allele of SNP rs356219)	58.49	52.0	0.59
*STK39* (A allele of SNP rs4668049)	27.45	39.13	0.32

Demographic, clinical and biochemical data of patients with Parkinson’s disease non-demented (PDND) and Parkinson’s disease with dementia (PDD) are presented with median (range) or percentage of total. *P*-values were determined using the Wilcoxon rank sum test or the Fisher’s exact test. Aao, age at onset; *ApoE4,* at least one *Apolipoprotein E4* allele; BDI, Beck Depression Inventory; CSF, cerebrospinal fluid; f, female; *GSK3B, Glycogen synthase kinase 3 beta*; *HLA-DRA, Human leucocyte antigen/Major histocompatibility complex, class II, DR alpha chain; HSPA5, Heat shock 70 kDa protein 5*; H&Y, Hoehn & Yahr stage; MMSE, Mini-Mental State Examination; p-tau, phospho-tau; *SNCA, Synuclein alpha; STK39, Serine threonine kinase 39*; t-tau, total tau; UPDRS, Unified Parkinson’s Disease Rating Scale.

### Cerebrospinal Fluid and Serum Collection

Serum collection was performed according to standardized protocols, for details see [33]. In brief, blood was centrifuged (2000 g, 4°C, 10 min) and stored at −80°C within 60 minutes after collection until analysis. Of patients who also underwent lumbar puncture, only those with normal routine CSF diagnostics were included (n = 36); slightly increased CSF albumin levels (up to 450 mg/L) were tolerated. CSF was also centrifuged (4000 g, 4°C, 10 min) and stored at −80°C within 60 min after collection. Spinal taps were only performed in patients but not in controls due to the invasive nature of CSF sampling.

### NAb ELISAs

The detailed process of ELISA development and measurement procedures has been described previously [13], [34]. In brief, for quantitative determination of NAbs against Abeta_1–42_, Asyn, MBP, MOG, S100B, and scrambled Abeta_1–40_ (for assessment of unspecific binding) high binding microtiter plates were coated with commercially available recombinant proteins expressed in *E. coli* (non-modified Abeta_1–42_, non-modified Asyn, and scrambled Abeta_1–40_ with the amino acid sequence KVKGLIDGAHIGDLVYEFMASNSFREGVGAGHVHVAQVEF, all from rPeptide, Bogart, Georgia GA, USA) or purified native protein (human S100B, Cell Sciences, Canton, Massachusetts MA, USA; bovine MBP and MOG, GMBU eV, Halle, Germany). Optimal concentration for protein immobilization was determined for each protein individually. Thus, 100 µl of a 5 mg/L Abeta_1–42_, 1 mg/L Asyn, 10 mg/L MBP, 10 mg/L MOG, and 1 mg/L S100B solution, respectively, were incubated in a well of a high binding microtiter plate (Corning Inc, Lowell, Massachusetts MA, USA) overnight at room temperature. For control purposes [35] microtiter plates were coated with 1 mg/L scrambled Abeta_1–40_ [36]. Non-specific binding was blocked by an ensuing incubation with 1% BSA in phosphate buffered saline with a pH of 7.4. After washing and drying, plates were incubated with diluted human serum samples under agitation at 350 rpm for 2 h at room temperature. The biotinylated detection antibody and horseradish-peroxidase streptavidin-conjugate were then added sequentially. Each was incubated for 60 min at room temperature. After the addition of substrate and stop solution, optical densities of the reactions were measured at 450 nm. For specificity reasons each sample was also applied to uncoated wells and the resulting unspecific signal was subtracted from the signal of the wells coated with the antigen. In addition, all respective scrambled Abeta_1–40_ NAb titres were subtracted from individual NAb titres. For quantification, an internal serum standard was applied and arbitrary titre units were calculated. All samples were measured within the same test kit lot. Inter-assay variability within this study was measured by applying four control samples and revealed a variation of 9; 12; 13; 10; 12 and 15% for Abeta_1–42_, Asyn, MBP, MOG, S100B, and scrambled Abeta_1–40_, respectively. The analytical sensitivities, defined as the mean NAb titre of the zero sample +3 SD, were 0.52; 0.35; 0.48; 0.30; 0.83 arbitrary titre units for Abeta_1–42_, Asyn, MBP, MOG, and S100B specific NAbs (n ≥12), respectively.

All NAb ELISA measurements were performed at Mediagnost, Reutlingen, Germany, blinded to clinical and demographic data.

### Abeta_1–42_, Total Tau and Phospho-tau Measurement

CSF Abeta_1–42_, total tau and phospho-tau levels were determined using commercially available ELISA kits (Innogenetics NV, Ghent, Belgium). We did not determine Asyn levels because we did not have access to CSF of controls, and the value of Asyn levels in blood for the differentiation of PD from controls is very probably low [12].

### SNP and ApoE Genotyping

Genomic DNA was extracted using standard protocols. SNPs located at the exon (rs429358 and rs7412 in exon 4 of the *APOE* gene), the intron (rs6438552 of *GSK3B*, rs3129882 of *HLA-DRA*, rs430397 of *HSPA5*, rs4668049 of *STK39*) or the untranslated region (rs356219 in the 3′-UTR of *SNCA*) were analyzed by the SNaPshot method. Briefly, a PCR with specific SNaPshot primers was performed. After single base extension with fluorescent-labelled ddNTPs the SNaPshot product was analyzed by capillary gel electrophoresis with ABI Prism 3100 Genetic Analyzer sequencer (Applied Biosystems Life Technologies GmbH, Darmstadt, Germany). Fluorescence data were analyzed using Gene Mapper™ Software v3.5, Applied Biosystems, Foster City, California CA, USA). All SNPs investigated were in Hardy-Weinberg equilibrium.

### Data Analysis

Data were analyzed with JMP software (version 9.0.2, SAS Institute Inc., Cary, North Carolina NC, USA). Demographic and clinical data are generally presented with the median and range (continuous data), or percentage of total (dichotomous data). *P*-values were obtained with the Wilcoxon rank sum or the Fisher’s exact test (PD versus controls; PD non-demented (PDND) versus PDD). NAb titres were logarithmized due to non-normal distribution. Associations of NAb titres with demographic, clinical or neurochemical data were calculated using multiple linear regression models, with age and gender (PD versus controls), and age, gender, age at onset and disease duration (PDND vs. PDD) as covariates. Differences were considered significant at *p*<0.05. Post-hoc analyses were corrected for multiple testing (i.e. 0.05 divided by the number of groups, see Table S1, S2, S3).

## Results

### Serum NAb Titres and Demographic Variables

Serum NAb titres of controls were not age-related (Abeta_1–42_, r^2^ = 0.00, *p* = 0.96; Asyn, r^2^ = 0.00, *p* = 0.74; MBP, r^2^ = 0.00, *p* = 0.76; MOG, r^2^ = 0.01, *p* = 0.16; S100B, r^2^ = 0.00, *p* = 0.53) and were comparable between females and males (0.19<*p*<0.92).

### Serum NAb Titres in Association with Diagnosis and Disease-associated Parameters

An overview of the association between NAbs and diagnosis is given in Table 3 and Table 4. Serum NAb titres differed neither significantly between PD patients and controls, nor between PDND and PDD patients. None of the NAb titres correlated significantly with demographic or clinical parameters (age at onset of parkinsonism, duration of parkinsonism, Hoehn & Yahr stage, BDI, and MMSE score; see also Table S1).

**Table 3:** Association of serum NAb titres with diagnosis (PD patients versus Controls).

Part A			
	PD	Controls	*p*-value
Abeta_1–42_ NAb (OD)	8.43 (0–11)	8.58 (0–10.98)	0.75
Asyn NAb	6.73 (0–9.46)	6.46 (0–10.80)	0.50
MBP NAb	7.76 (0–10.29)	7.94 (0–10.72)	0.98
MOG NAb	0 (0–8.86)	0 (0–9.51)	0.25
S100B NAb	8.01 (0–11.08)	8.11 (0–11.40)	0.46

Logarithmized serum NAb titres were calculated using a multiple linear regression model with age, gender (PD versus controls) as covariates. Data are presented with median (range). *P*-values <0.025 (0.05/2) were considered significant. Abeta_1–42_, Amyloid-beta_1–42_; Asyn, Alpha-synuclein; MBP, Myelin basic protein; MOG, Myelin oligodendrocyte glycoprotein; NAb, naturally occurring autoantibody; OD, Optical density; PD, Parkinson’s disease; S100B, S100 calcium binding protein B.

**Table 4:** Association of serum NAb titres with diagnosis (PDND patients versus PDD patients).

	PDND	PDD	*p*-value
Abeta_1–42_ NAb (OD)	8.54 (0–10.6)	8.23 (0–11)	0.14
Asyn NAb	7.17 (0–9.46)	6.0 (0–9.06)	0.33
MBP NAb	7.90 (0–9.87)	7.64 (0–10.29)	0.03
MOG NAb	0 (0–8.86)	0 (0–8.64)	0.72
S100B NAb	7.92 (0–11.08)	8.10 (5.95–9.52)	0.04

Logarithmized serum NAb titres were calculated using a multiple linear regression model with age, gender, age at onset parkinsonism and disease duration parkinsonism as covariates. Data are presented with median (range). *P*-values <0.025 (0.05/2) were considered significant. Abeta_1–42_, Amyloid-beta_1–42_; Asyn, Alpha-synuclein; MBP, Myelin basic protein; MOG, Myelin oligodendrocyte glycoprotein; NAb, naturally occurring autoantibody; OD, Optical density; PDD, Parkinson’s disease with dementia; PDND, Parkinson’s disease non-demented; S100B, S100 calcium binding protein B.

### Serum NAb Titres and Neurodegenerative Markers

No significant correlation between the serum NAb titres, and CSF Abeta_1–42_, total tau and p-tau was observed (Table S2).

### Serum NAb Titres and SNPs

Table S3 gives an overview of the associations between the NAb titres and SNPs assessed in this study (*APOE, GSK3B, HLA-DRA, HSPA5*, *SNCA,* and *STK39*). We did not find a relevant interaction between these variables.

## Discussion

This study indicates that neurodegeneration- and neuroinflammation-associated serum NAbs as used in this large cross-sectional clinical trial are not useful as trait and state markers in PD. They also do not seem to be determined by genetic variants associated with neurodegeneration and neuroinflammation in PD, and are not significantly associated with neurodegenerative markers in the CSF.

Our results are, in particular with regard to Asyn NAbs, partly confirmed by others [10], [11] but there are also studies reporting higher [9], [10] and lower [12] Asyn NAb levels in the blood of PD patients compared to that of controls. Papachroni *et al.* [11] examined serum of different kinds of Parkinsonism by immunoblot detection with denaturated recombinant human alpha-, beta- and gamma-synucleins. They found that multiepitopic autoantibodies against Asyn (this term corresponds to “naturally occurring autoantibodies” as used in this manuscript) are detectable in 65% of all patients tested and their presence strongly correlated with an inherited mode of PD but not with other disease-related factors. In more detail, the frequency of autoantibodies was comparable between patients with the sporadic form of PD and controls but was clearly increased in patients with familial form of the disease [11]. Both study cohort (e.g., mean age at examination 65 vs. 70 years, age at onset 60 vs. 62 years, H&Y stage 2.5 vs. 2) and results (no significant differences between idiopathic PD and controls) are basically comparable with the results of this study.

Gruden *et al.* [10] found elevated titres of NAbs against Asyn monomers, oligomers and fibrils in 72%, 56%, and 17% in the serum of PD patients, which reached a maximum approximately 5 years after diagnosis and were markedly lower 10 years after diagnosis. The control cohort showed autoantibody titres against Asyn, dopamine and S100B which were comparable with levels of PD patients with a mean disease duration of 10 years. As our study cohort has a mean disease duration of 9.5 years, our results basically confirm this result of Gruden and colleagues. Moreover, as we have a cross-sectional design in this study, we cannot exclude that patients with PD, as shown in this previous study, have increased values of respective NAbs at earlier disease stages.

Yanamandra *et al.* [9] found significantly higher autoantibody levels towards monomeric Asyn in the serum of patients with PD with a mean disease duration of 7.7 years, compared to controls using ELISA and Western Blot. Interestingly, also in this study PD patients with longer disease duration (9.9 years) had a lower immune response than those with shorter disease duration (5.9 years, groups divided by use of the H&Y scale). The weakness of this study is the low sample size however the (repeated) observation that levels of respective autoantibodies may be particularly high at early disease stages requires further investigation. Notably, we did not find any relevant correlation between respective autoantibody titres and disease duration in our PD cohort.

In the analyses of Besong-Agbo *et al.* [12] levels of NAbs against Asyn were significantly lower in patients with PD compared to healthy controls and patients with AD. Comparable to our study cohort, their study included patients with rather advanced stages of PD (mean disease duration 10.2 years, 80% with a H&Y stage ≥2). Study cohorts are obviously comparable to our actual study, thus differences observed between the two studies are not well explained by demographic and clinical differences of the cohorts. We also used comparable techniques to consider technical issues (e.g. background staining, unspecific binding). Thus, differences are most probably due to the inclusion of different PD phenotypes, indicating that future research should particularly focus on the definition of potentially existing endophenotypes of PD which may be influenced by immune responses. Interestingly, Besong-Agbo *et al.* did not detect any relevant association of autoantibody levels with demographic and clinical variables, including disease duration, which is comparable to results obtained in this study. However, here we could not replicate a pilot study performed by our group, in which we found higher NAb titres, including NAb titres against Asyn, in a small sample of PDD and DLB patients (no overlap with the cohort reported here), than in other dementias and in controls [13].

In addition to the lack of differences between NAb titres of individuals with or without PD, NAb titres in this study were neither relevantly associated with disease-associated clinical parameters (such as H&Y stage and dementia), nor neurochemical markers or SNPs which were shown to influence the inflammation or amyloid cascade. At this point it is difficult to explain these discrepancies between our actual study and previous investigations, however we consider the following aspects: (i) This is the by far largest PD cohort studied up to now on this topic, including patients from the early to very advanced stages (Hoehn & Yahr 1–4), and with a specific focus on PD patients with cognitive deficits. As we did not find any relevant associations of NAbs with disease occurrence and additional disease-associated parameters in this study, it is most probable that this cohort reflects the general situation of PD better, than did the pilot study. (ii) This discrepancy as well as differing results from the previously mentioned studies may however also indicate that subcohorts - or endophenotypes - among the Lewy body disorders spectrum exist which have indeed a (pathophysiologically) relevant change in NAb levels. (iii) It was hypothesized that the low avidity of the multiepitopic NAbs can lead to problems in differentiating the antibody binding signal from unspecific background activity [12], [37]. We addressed this issue by using a specific, biotinylated goat-anti-human-IgG binding system in combination with a streptavidin-peroxidase conjugate. Moreover, all results were corrected for unspecific binding with regard to both, coated minus uncoated well and specific minus unspecific (individual NAbs minus scrambled Abeta_1–40_) detection antibodies.

In this study, we did not differentiate between free and loaded autoantibody titres. It is indeed probable that, in a physiological environment, the majority of NAbs are bound to self-antigens [38] and are thus not or hardly detectable by current assessment strategies [39]. Future studies should address this aspect however it may be difficult to measure total amount of respective antibodies as they can change their binding characteristics under conditions which lead to release of the antigen-antibody complex [37].

In conclusion, it must be considered that recombinant as well as purified metabolites may not be ideal for delineating NAb titres in physiological biofluids, and that the use of altered proteins and peptides as antigens may have more potential to detect disease-specific changes of autoantibody levels [40].

## Conclusion

These results argue against a relevant potential of serum NAb titres to serve as trait or state markers in PD.

## Supporting Information

Table S1 **Association of serum NAb titres with demographic and clinical parameters.** *P*-values of NAb titres were calculated using a multiple linear regression model, corrected for age at onset of parkinsonism, disease duration of parkinsonism, Hoehn & Yahr stage, age at onset of dementia, and MMSE. *P*-values <0.01 (0.05/5) were considered significant. Abeta_1–42_, Amyloid-beta_1–42_; Asyn, Alpha-synuclein; H&Y, Hoehn & Yahr stage; MBP, Myelin basic protein; MMSE, Mini-Mental State Examination; MOG, Myelin oligodendrocyte glycoprotein; NAb, naturally occuring autoantibody; PD, Parkinson’s disease; S100B, S100 calcium binding protein B.
(DOC)

Table S2 **Association of serum NAb titres with neurodegenerative markers.** Neurodegenerative markers (CSF Abeta_1–42_, t-tau and p-tau) are analyzed as described. NAb titres of scrambled Abeta_1–40_ were subtracted from other NAb titres, and were logarithmized afterwards. *P*-values of NAb titres were calculated using the Spearmans non-parametric correlation. Post-hoc analyses were performed for comparison of NAb titres and neurodegenerative marker levels between the following cohorts: PD patients versus controls, Parkinson’s disease non-demented (PDND) versus Parkinson’s disease with dementia (PDD). *P*-values <0.0025 (0.05/20) were considered significant. Abeta_1–42_, Amyloid-beta_1–42_; Asyn, Alpha-synuclein; CSF, cerebrospinal fluid; MBP, Myelin basic protein; MOG, Myelin oligodendrocyte glycoprotein; NAb, naturally occuring autoantibody; PD, Parkinson’s disease; PDD, Parkinson’s disease with dementia; PDND, Parkinson’s disease non-demented; p-tau, phospho-tau; S100B, S100 calcium binding protein B; t-tau, total-tau.
(DOC)

Table S3 **P-values of serum NAb titres and SNPs.** For Single nucleotide polymorphisms (SNPs) the respective gene name and reference SNP ID number (rs#) are given. NAb titres of scrambled Abeta_1–40_ were subtracted from other NAb titres, and were logarithmized afterwards. *P*-values of NAb titres were calculated using the Wilcoxon signed rank test/Chi^2^ approximation. Post-hoc analyses were performed for comparison of NAb titres and single nucleotide polymorphisms between the following cohorts: PD patients versus controls, Parkinson’s disease non-demented (PDND) versus Parkinson’s disease with dementia (PDD). *P*-values <0.0025 (0.05/20) were considered significant. Abeta_1–42_, Amyloid-beta_1–42_; *Apo E4, Apolipoprotein E4*; Asyn, Alpha-synuclein; *GSK3B, Glycogen synthase kinase 3 beta*; *HLA-DRA, Human leucocyte antigen/Major histocompatibility complex, class II, DR alpha chain*; *HSPA5, Heat shock 70 kDa protein 5;* MBP, Myelin basic protein; MOG, Myelin oligodendrocyte glycoprotein; NAb, naturally occuring autoantibody; PD, Parkinson’s disease; PDD, Parkinson’s disease with dementia; PDND, Parkinson’s disease non-demented; S100B, S100 calcium binding protein B; *SNCA, Synuclein alpha; STK39, Serine threonine kinase 39*.
(DOC)
