# Supplementary material for: Comparable Autoantibody Serum Levels against Amyloid- and Inflammation-Associated Proteins in Parkinson’s Disease Patients and Controls
Source: PLoS One. 2014 Feb 21;9(2):e88604. doi: 10.1371/journal.pone.0088604 (PMC3931625; doi:10.1371/journal.pone.0088604)
Supplement: Table S1 — Association of serum NAb titres with demographic and clinical parameters. P-values of NAb titres were calculated using a multiple linear regression model, corrected for age at onset of parkinsonism, disease duration of parkinsonism, Hoehn & Yahr stage, age at onset of dementia, and MMSE. P-values <0.01 (0.05/5) were considered significant. Abeta1–42, Amyloid-beta1–42; Asyn, Alpha-synuclein; H&Y, Hoehn & Yahr stage; MBP, Myelin basic protein; MMSE, Mini-Mental State Examination; MOG, Myelin oligodendrocyte glycoprotein; NAb, naturally occuring autoantibody; PD, Parkinson’s disease; S100B, S100 calcium binding protein B. (DOC) [file pone.0088604.s001.doc]

**Table S1: Association of serum NAb titres with demographic and clinical parameters**

|  | PD |
| --- | --- |
| Age at onset parkinsonism | |
| Abeta_1-42_ NAb | 0.59 |
| Asyn NAb | 0.24 |
| MBP NAb | 0.26 |
| MOG NAb | 0.09 |
| S100B NAb | 0.91 |
| Duration parkinsonism | |
| Abeta_1-42_ NAb | 0.61 |
| Asyn NAb | 0.81 |
| MBP NAb | 0.26 |
| MOG NAb | 0.08 |
| S100B NAb | 0.99 |
| H&Y stage | |
| Abeta_1-42_ NAb | 0.32 |
| Asyn NAb | 0.48 |
| MBP NAb | 0.21 |
| MOG NAb | 0.09 |
| S100B NAb | 0.72 |
| Age at onset dementia | |
| Abeta_1-42_ NAb | 0.65 |
| Asyn NAb | 0.16 |
| MBP NAb | 0.32 |
| MOG NAb | 0.08 |
| S100B NAb | 0.88 |
| MMSE | |
| Abeta_1-42_ NAb | 0.59 |
| Asyn NAb | 0.17 |
| MBP NAb | 0.76 |
| MOG NAb | 0.07 |
| S100B NAb | 0.84 |

*P*-values of NAb titres were calculated using a multiple linear regression model, corrected for age at onset of parkinsonism, disease duration of parkinsonism, Hoehn & Yahr stage, age at onset of dementia, and MMSE. *P*-values < 0.01 (0.05/5) were considered significant. Abeta_1-42_, Amyloid-beta_1-42_; Asyn, Alpha-synuclein; H&Y, Hoehn & Yahr stage; MBP, Myelin basic protein; MMSE, Mini-Mental State Examination; MOG, Myelin oligodendrocyte glycoprotein; NAb, naturally occuring autoantibody; PD, Parkinson`s disease; S100B, S100 calcium binding protein B.
