# Supplementary material for: Comparable Autoantibody Serum Levels against Amyloid- and Inflammation-Associated Proteins in Parkinson’s Disease Patients and Controls
Source: PLoS One. 2014 Feb 21;9(2):e88604. doi: 10.1371/journal.pone.0088604 (PMC3931625; doi:10.1371/journal.pone.0088604)
Supplement: Table S2 — Association of serum NAb titres with neurodegenerative markers. Neurodegenerative markers (CSF Abeta1–42, t-tau and p-tau) are analyzed as described. NAb titres of scrambled Abeta1–40 were subtracted from other NAb titres, and were logarithmized afterwards. P-values of NAb titres were calculated using the Spearmans non-parametric correlation. Post-hoc analyses were performed for comparison of NAb titres and neurodegenerative marker levels between the following cohorts: PD patients versus controls, Parkinson’s disease non-demented (PDND) versus Parkinson’s disease with dementia (PDD). P-values <0.0025 (0.05/20) were considered significant. Abeta1–42, Amyloid-beta1–42; Asyn, Alpha-synuclein; CSF, cerebrospinal fluid; MBP, Myelin basic protein; MOG, Myelin oligodendrocyte glycoprotein; NAb, naturally occuring autoantibody; PD, Parkinson’s disease; PDD, Parkinson’s disease with dementia; PDND, Parkinson’s disease non-demented; p-tau, phospho-tau; S100B, S100 calcium binding protein B; t-tau, total-tau. (DOC) [file pone.0088604.s002.doc]

**Table S1: Association of serum NAb titres with neurodegenerative markers**

|  | PD | PDND | PDD |
| --- | --- | --- | --- |
| CSF Abeta_1-42_ | | | |
| Abeta_1-42_ NAb | 0.20 | 0.003 | 0.89 |
| Asyn NAb | 0.88 | 0.14 | 0.18 |
| MBP NAb | 0.78 | 0.79 | 0.13 |
| MOG NAb | 0.87 | 0.16 | 0.24 |
| S100B NAb | 0.16 | 0.51 | 0.46 |
| CSF t-tau | | | |
| Abeta_1-42_ NAb | 0.83 | 0.81 | 0.74 |
| Asyn NAb | 0.04 | 0.27 | 0.07 |
| MBP NAb | 0.61 | 0.63 | 0.68 |
| MOG NAb | 0.13 | 0.52 | 0.30 |
| S100B NAb | 0.63 | 0.17 | 0.93 |
| CSF p-tau | | | |
| Abeta_1-42_ NAb | 0.43 | 0.99 | 0.34 |
| Asyn NAb | 0.31 | 0.93 | 0.39 |
| MBP NAb | 0.57 | 0.55 | 0.80 |
| MOG NAb | 0.04 | 0.16 | 0.27 |
| S100B NAb | 0.73 | 0.23 | 0.72 |

Neurodegenerative markers (CSF Abeta_1-42_, t-tau and p-tau) are analyzed as described.

NAb titres of scrambled Abeta_1-40_ were subtracted from other NAb titres, and were logarithmized afterwards. *P*-values of NAb titres were calculated using the Spearmans non-parametric correlation. Post-hoc analyses were performed for comparison of NAb titres and neurodegenerative marker levels between the following cohorts: PD patients versus controls, Parkinson’s disease non-demented (PDND) versus Parkinson’s disease with dementia (PDD). *P*-values < 0.0025 (0.05/20) were considered significant. Abeta_1-42_, Amyloid-beta_1-42_; Asyn, Alpha-synuclein; CSF, cerebrospinal fluid; MBP, Myelin basic protein; MOG, Myelin oligodendrocyte glycoprotein; NAb, naturally occuring autoantibody; PD, Parkinson`s disease; PDD, Parkinson’s disease with dementia; PDND, Parkinson’s disease non-demented; p-tau, phospho-tau; S100B, S100 calcium binding protein B; t-tau, total-tau.
