# Supplementary material for: Comparable Autoantibody Serum Levels against Amyloid- and Inflammation-Associated Proteins in Parkinson’s Disease Patients and Controls
Source: PLoS One. 2014 Feb 21;9(2):e88604. doi: 10.1371/journal.pone.0088604 (PMC3931625; doi:10.1371/journal.pone.0088604)
Supplement: Table S3 — P-values of serum NAb titres and SNPs. For Single nucleotide polymorphisms (SNPs) the respective gene name and reference SNP ID number (rs#) are given. NAb titres of scrambled Abeta1–40 were subtracted from other NAb titres, and were logarithmized afterwards. P-values of NAb titres were calculated using the Wilcoxon signed rank test/Chi2 approximation. Post-hoc analyses were performed for comparison of NAb titres and single nucleotide polymorphisms between the following cohorts: PD patients versus controls, Parkinson’s disease non-demented (PDND) versus Parkinson’s disease with dementia (PDD). P-values <0.0025 (0.05/20) were considered significant. Abeta1–42, Amyloid-beta1–42; Apo E4, Apolipoprotein E4; Asyn, Alpha-synuclein; GSK3B, Glycogen synthase kinase 3 beta; HLA-DRA, Human leucocyte antigen/Major histocompatibility complex, class II, DR alpha chain; HSPA5, Heat shock 70 kDa protein 5; MBP, Myelin basic protein; MOG, Myelin oligodendrocyte glycoprotein; NAb, naturally occuring autoantibody; PD, Parkinson’s disease; PDD, Parkinson’s disease with dementia; PDND, Parkinson’s disease non-demented; S100B, S100 calcium binding protein B; SNCA, Synuclein alpha; STK39, Serine threonine kinase 39. (DOC) [file pone.0088604.s003.doc]

**Table S1: *P*-values of serum NAb titres and SNPs**

|  | All | Controls | PD | PDND | PDD |
| --- | --- | --- | --- | --- | --- |
| At least 1 *ApoE4* allele | | | | | |
| Abeta_1-42_ NAb | 0.13 | 0.17 | 0.47 | 0.89 | 0.19 |
| Asyn NAb | 0.07 | 0.04 | 0.81 | 0.90 | 0.81 |
| MBP NAb | 0.72 | 0.80 | 0.69 | 0.52 | 0.93 |
| MOG NAb | 0.76 | 0.62 | 0.81 | 1 | 0.88 |
| S100B NAb | 0.61 | 0.76 | 0.71 | 0.69 | 0.81 |
| *GSK3B* (SNP rs6438552) | | | | | |
| Abeta_1-42_ NAb | 0.73 | 0.42 | 0.24 | 0.91 | 0.09 |
| Asyn NAb | 0.96 | 0.83 | 0.64 | 0.37 | 0.41 |
| MBP NAb | 0.39 | 0.32 | 0.53 | 0.40 | 0.47 |
| MOG NAb | 0.85 | 0.97 | 0.68 | 0.35 | 0.50 |
| S100B NAb | 0.83 | 0.67 | 0.59 | 0.14 | 0.41 |
| *HLA-DRA* (SNP rs3129882) | | | | | |
| Abeta_1-42_ NAb | 0.62 | 0.52 | 0.82 | 0.41 | 0.11 |
| Asyn NAb | 0.79 | 0.82 | 0.39 | 0.65 | 0.18 |
| MBP NAb | 0.18 | 0.11 | 0.80 | 0.50 | 0.15 |
| MOG NAb | 0.66 | 0.34 | 0.09 | 0.22 | 0.06 |
| S100B NAb | 0.47 | 0.46 | 0.90 | 0.94 | 0.82 |
| *HSPA5* (SNP rs430397) | | | | | |
| Abeta_1-42_ NAb | 0.43 | 0.18 | 0.58 | 0.61 | 0.95 |
| Asyn NAb | 0.50 | 0.50 | 0.16 | 0.08 | 0.77 |
| MBP NAb | 0.19 | 0.28 | 0.37 | 0.54 | 0.19 |
| MOG NAb | 0.03 | 0.09 | 0.06 | 0.26 | 0.09 |
| S100B NAb | 0.48 | 0.93 | 0.06 | 0.07 | 0.74 |
| *SNCA* (SNP rs356219) | | | | | |
| Abeta_1-42_ NAb | 0.23 | 0.36 | 0.59 | 0.82 | 0.16 |
| Asyn NAb | 0.77 | 0.80 | 0.90 | 0.70 | 0.96 |
| MBP NAb | 0.50 | 0.74 | 0.62 | 0.28 | 0.70 |
| MOG NAb | 0.89 | 0.64 | 0.76 | 0.30 | 0.49 |
| S100B NAb | 0.80 | 0.70 | 0.34 | 0.16 | 0.36 |
| *STK39* (SNP rs4668049) | | | | | |
| Abeta_1-42_ NAb | 0.38 | 0.37 | 0.08 | 0.10 | 0.16 |
| Asyn NAb | 0.23 | 0.63 | 0.17 | 0.17 | 0.20 |
| MBP NAb | 0.85 | 0.91 | 0.87 | 0.21 | 0.50 |
| MOG NAb | 0.62 | 0.91 | 0.48 | 0.58 | 0.62 |
| S100B NAb | 0.06 | 0.16 | 0.20 | 0.62 | 0.02 |

For Single nucleotide polymorphisms (SNPs) the respective gene name and reference SNP ID number (rs#) are given. NAb titres of scrambled Abeta_1-40_ were subtracted from other NAb titres, and were logarithmized afterwards. *P*-values of NAb titres were calculated using the Wilcoxon signed rank test/Chi^2^ approximation. Post-hoc analyses were performed for comparison of NAb titres and single nucleotide polymorphisms between the following cohorts: PD patients versus controls, Parkinson’s disease non-demented (PDND) versus Parkinson’s disease with dementia (PDD). *P*-values < 0.0025 (0.05/20) were considered significant. Abeta_1-42_, Amyloid-beta_1-42_; *Apo E4, Apolipoprotein E4*; Asyn, Alpha-synuclein; *GSK3B, Glycogen synthase kinase 3 beta*; *HLA-DRA, Human leucocyte antigen/Major histocompatibility complex, class II, DR alpha chain*; *HSPA5, Heat shock 70 kDa protein 5;* MBP, Myelin basic protein; MOG, Myelin oligodendrocyte glycoprotein; NAb, naturally occuring autoantibody; PD, Parkinson`s disease; PDD, Parkinson’s disease with dementia; PDND, Parkinson’s disease non-demented; S100B, S100 calcium binding protein B; *SNCA, Synuclein alpha; STK39, Serine threonine kinase 39*.
